# A Low-Cost Hydrogel Electrode for Multifunctional Sensing: Strain, Temperature, and Electrophysiology

**DOI:** 10.3390/bios15030177

**Published:** 2025-03-11

**Authors:** Junjie Zheng, Jinli Zhou, Yixin Zhao, Chenxiao Wang, Mengzhao Fan, Yunfei Li, Chaoran Yang, Hongying Yang

**Affiliations:** 1 College of Intelligent Textile and Fabric Electronics, Zhongyuan University of Technology, Zhengzhou 450007, China; 2022110445@zut.edu.cn (J.Z.); 19139830666@163.com (Y.Z.); 2023110482@zut.edu.cn (C.W.); fmz811600@gmail.com (M.F.); liyunfei@zut.edu.cn (Y.L.); 2022110441@zut.edu.cn (C.Y.); 2 Henan Province Collaborative Innovation Center of Textile and Garment Industry, Zhengzhou 450007, China

**Keywords:** multifunctional sensors, strain, temperature, hydrogel, electrophysiological signals

## Abstract

With the rapid development of wearable technology, multifunctional sensors have demonstrated immense application potential. However, the limitations of traditional rigid materials restrict the flexibility and widespread adoption of such sensors. Hydrogels, as flexible materials, provide an effective solution to this challenge due to their excellent stretchability, biocompatibility, and adaptability. This study developed a multifunctional flexible sensor based on a composite hydrogel of polyvinyl alcohol (PVA) and sodium alginate (SA), using poly(3,4-ethylenedioxythiophene)/polystyrene sulfonate (PEDOT:PSS) as the conductive material to achieve multifunctional detection of strain, temperature, and physiological signals. The sensor features a simple fabrication process, low cost, and low impedance. Experimental results show that the prepared hydrogel exhibits outstanding mechanical properties and conductivity, with a strength of 118.8 kPa, an elongation of 334%, and a conductivity of 256 mS/m. In strain sensing, the sensor demonstrates a rapid response to minor strains (4%), high sensitivity (gauge factors of 0.39 for 0–120% and 0.73 for 120–200% strain ranges), short response time (2.2 s), low hysteresis, and excellent cyclic stability (over 500 cycles). For temperature sensing, the sensor achieves high sensitivities of −27.43 Ω/K (resistance mode) and 0.729 mV/K (voltage mode), along with stable performance across varying temperature ranges. Furthermore, the sensor has been successfully applied to monitor human motion (e.g., finger bending, wrist movement) and physiological signals such as electrocardiogram (ECG), electromyogram (EMG), and electroencephalogram (EEG), highlighting its significant potential in wearable health monitoring. By employing a simple and efficient fabrication method, this study presents a high-performance multifunctional flexible sensor, offering novel insights and technical support for the advancement of wearable devices.

## 1. Introduction

In recent years, with the rapid development of wearable devices and intelligent sensing technologies, there has been a growing demand for low-cost, high-performance multifunctional sensors in fields such as health monitoring [1], motion tracking [2], and human–machine interface applications [3]. However, the fabrication of sensors using rigid materials faces numerous challenges, including high costs, complex manufacturing processes, and functional limitations. In contrast, hydrogel-based multifunctional sensors offer advantages in terms of fabrication simplicity, cost efficiency, and flexibility. These sensors can detect multiple signals simultaneously, such as temperature [4,5], strain [6,7], and electrophysiological signals [8] (including electrocardiography (ECG) [9,10], electromyography (EMG) [11,12], and electroencephalography (EEG) [13,14]), providing comprehensive health monitoring data. As such, they have become a key focus in current research.

Conductive hydrogels, as flexible materials, have demonstrated extensive potential in sensor applications due to their excellent mechanical properties and biocompatibility [15,16]. Their flexibility gives conductive hydrogels significant advantages in strain sensors, allowing the real-time monitoring of dynamic human movements [17], such as joint motion and muscle activities. Furthermore, conductive hydrogels have been widely and effectively applied in electrophysiological signal monitoring, particularly for capturing ECG, EMG, and EEG signals, where they exhibit exceptional skin conformity [18]. The skin-friendly nature of hydrogels enables them to effectively fill the gaps between skin pores, significantly reducing contact impedance and mitigating motion artifacts during ECG monitoring [19]. In EMG signal monitoring, hydrogels can precisely track muscle activity [20], offering critical clinical significance in muscle injury assessment. Additionally, hydrogels are highly sensitive to EEG signal fluctuations, aiding in brain activity monitoring [21].

Polyvinyl alcohol (PVA) is a commonly used base material for hydrogels. Due to its hydrophilic hydroxyl (-OH) groups, PVA can form robust hydrogel structures through physical or chemical crosslinking [22], such as freeze–thaw cycling [23] or borate crosslinking [24]. Meanwhile, sodium alginate (SA) is widely utilized in biomedical fields due to its excellent biocompatibility and antibacterial properties [25]. SA contains abundant carboxyl (-COOH) and hydroxyl (-OH) groups, which can form flexible networks with PVA through hydrogen bonding and coordination between carboxyl groups and metal ions [26,27]. However, hydrogels fabricated solely through PVA and SA interactions often fail to meet the demands for high conductivity. To address this issue, conductive fillers such as carbon-based nanomaterials, metal nanoparticles, or conductive polymers are typically introduced to enhance hydrogel conductivity [28]. While carbon-based and metal nanomaterials exhibit outstanding conductivity, their high costs and limited processability restrict their widespread application. In contrast, conductive polymers, particularly poly(3,4-ethylenedioxythiophene):polystyrene sulfonate (PEDOT:PSS), have emerged as key components in conductive hydrogels due to their excellent conductivity, flexibility, and processability [29]. In PEDOT:PSS, the PSS dopant interacts with the conjugated PEDOT backbone to generate charges that migrate along the conjugated chain. PSS not only ensures charge neutrality but also facilitates effective charge transport through electrostatic interactions and chain conformation optimization. Three primary molecular interactions exist within PEDOT:PSS: (i) electrostatic attraction between the π-conjugated PEDOT chains and the negatively charged PSS chains, (ii) π–π stacking among adjacent PEDOT chains, and (iii) chain entanglements primarily formed among the long PSS chains [30]. These interactions contribute to the production of PEDOT:PSS-based conductive hydrogels with high conductivity, mechanical strength, and uniform dispersion. Furthermore, as a highly conductive, temperature-sensitive material [31], PEDOT:PSS enables temperature sensing through carrier hopping [32]. For example, Zhao et al. [33] developed a conductive hydrogel using PVA, PEDOT:PSS, and liquid metal nanoparticles, which exhibited superior self-healing and adhesive properties with strain sensing up to 80 kPa. Chai et al. [34] fabricated a PVA/PEDOT:PSS hydrogel with a high Seebeck coefficient of 1.31 mV/K, while Wang et al. [35] demonstrated the potential of a PVA/CMC/PEDOT:PSS hydrogel for stable ECG monitoring, providing a cost-effective alternative to traditional Ag/AgCl electrodes.

**Figure 1:**
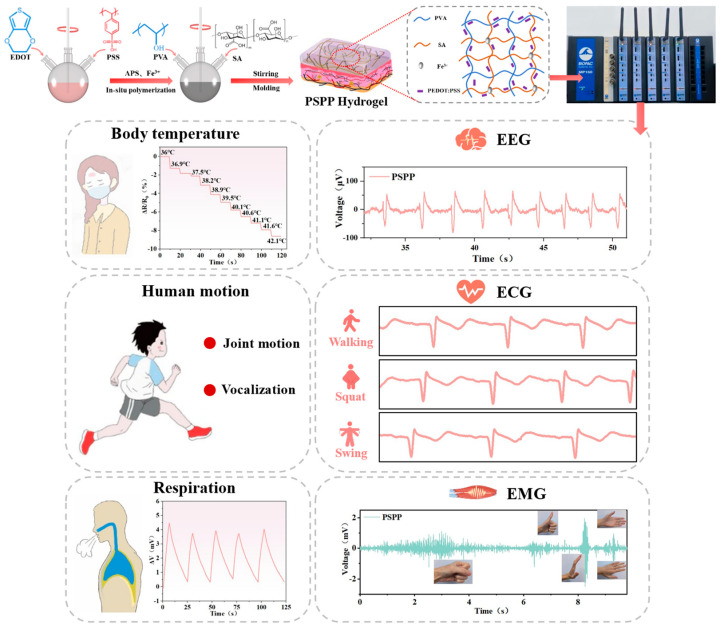
The PSPP hydrogel enables the sensing of human motion, temperature, and physiological signals.

This study employs PVA and SA as the primary network materials, combined with PEDOT:PSS as the conductive polymer, to fabricate a low-cost, highly flexible, and low-impedance PSPP hydrogel with multifunctional sensing capabilities using a one-pot freeze–thaw method. During synthesis, 3,4-ethylenedioxythiophene (EDOT) monomers and polystyrene sulfonate (PSS) were added to a three-neck flask with ammonium persulfate (APS) as the oxidizing agent and ferric chloride (FeCl_3_) as the catalyst to form PEDOT:PSS via oxidative polymerization. Subsequently, PVA and SA powders were added, completing the PSPP hydrogel synthesis. The hydrogen bond crosslinking formed during the freeze–thaw process provided physical entanglement points, while free Fe^3+^ ions from FeCl_3_ reacted with the carboxyl groups in SA molecules, further enhancing the hydrogel structure [36]. The PSPP hydrogel exhibits a tensile strength of 118.8 kPa, an elongation of 334%, and a conductivity of 256 mS/m. It demonstrates excellent strain-sensing performance, accurately responding to small strains (4%) with high stability. The hydrogel also shows outstanding temperature-sensing capabilities, achieving sensitivities of −27.43 Ω/K and 0.729 mV/K. Additionally, the PSPP hydrogel enables the monitoring of human joint movements, vocal cord vibrations, body temperature variations (e.g., fever), respiratory activity, touch operations (e.g., typing, dialing, and writing), and electrophysiological signals (ECG, EMG, and EEG) (Figure 1). These results highlight the PSPP hydrogel’s exceptional mechanical properties, sensing functionality, and broad application prospects, particularly in health monitoring and wearable device fields.

## 2. Materials and Methods

### 2.1. Materials

Polyvinyl alcohol (PVA-124) (AR, purchased from Xilong Scientific Co., Ltd., Guangzhou, China); SA (AR, purchased from Tianjin Biolun Biological Technology Co., Ltd., Tianjin, China); 3,4-ethylenedioxythiophene (EDOT) (99%, purchased from Macklin, Shanghai, China); poly(styrenesulfonic acid) (PSS) (Mw: 75,000, purchased from Macklin); ammonium persulfate (APS) and ferric chloride (FeCl_3_) (purchased from Shanghai Macklin Biochemical Technology Co., Ltd., Shanghai, China); deionized water: these were used in all experiments.

### 2.2. Preparation of PVA/SA/PEDOT:PSS Hydrogel

A specific amount of PSS, based on a mass ratio of 1:3 to EDOT, was dissolved in deionized water and stirred at room temperature for 2 h until the solution became clear and free of oily residues. The pH of the solution was then adjusted to 2–3, resulting in Solution A. Separately, APS was weighed at a mass ratio of 4:5, and FeCl_3_ at a mass ratio of 50:1, relative to their respective components. These were dissolved in deionized water with stirring to form Solution B. Solution A and Solution B were then mixed and stirred until the color of the mixture no longer changed. A certain amount of PVA and SA powders were added to the PEDOT:PSS solution and dissolved thoroughly to obtain a PVA/SA/PEDOT:PSS mixed solution. This solution was poured into a mold and frozen at −25 °C for 14 h, followed by thawing at room temperature for 8 h. The freeze–thaw process was repeated twice, resulting in the formation of PVA/SA/PEDOT:PSS (PSPP) hydrogel.

### 2.3. Characterization and Testing

All hydrogel samples were freeze-dried using a freeze-drying vacuum system (ZLGJ-18, Shanghai, China) and subsequently ground into powder using a mortar and pestle. The cross-sectional morphology and elemental distribution of the hydrogel samples were observed via field-emission scanning electron microscopy (SEM) and energy-dispersive spectroscopy (EDS). Functional groups and dynamic covalent bonds within the hydrogel samples were analyzed using a Fourier-transform infrared spectrometer (FTIR, Bruker Tensor 37e, Karlsruhe, Germany).

Electrochemical measurements were conducted with an electrochemical workstation (Chi-760E, Chenhua, Shanghai, China). Skin-contact impedance tests were performed using an impedance analyzer (IM3533-01 LCR, HIOKI, Ueda City, Japan). Strain-sensing tests were carried out using a flexible electronics multimodal testing system (ST600C, Shengte, Suzhou, China). Temperature-sensing tests were conducted using a digital source meter (Keithley 2450, Keithley Instruments, Cleveland, OH, USA) and a high-precision digital multimeter (VC86E, Victor, Shenzhen, China). Finally, ECG electrophysiological signal monitoring was performed using a physiological signal monitoring system (BIOPAC MP160, Biopac Systems Inc., Goleta, CA, USA).

### 2.4. Mechanical Properties of PSPP Hydrogels

At 25 °C, the mechanical properties of the PSPP hydrogel were evaluated using a flexible electronics multimodal testing system (ST600C, Suzhou, China). The hydrogel samples were precisely cut into rectangular dimensions of 10 mm × 5 mm × 2 mm and subjected to stretching at a rate of 20 mm/min.

### 2.5. Electrochemical Properties of PSPP Hydrogels

The I-T curve, I-V curve, and impedance of the PSPP hydrogel were tested using an electrochemical workstation (Chi-760E, Chenhua, Shanghai, China). The I-T curve was used to calculate the conductivity of the hydrogel. Before testing, the hydrogel was uniformly cut into dimensions of 10 mm × 2 mm × 2 mm, and excess surface moisture was absorbed using filter paper. The formula for calculating the hydrogel’s resistance is as follows:

(1) $$R=\frac{U}{I}$$

where *R* is the resistance of the hydrogel in ohms (Ω); *U* is the voltage set by the electrochemical workstation in volts (V); and *I* is the stabilized current in amperes (A). The formula for calculating the conductivity of the hydrogel is as follows:

(2) $$\sigma =\frac{L}{RS}$$

where *L* is the length of the sample in meters (m); *R* is the resistance of the sample in ohms (Ω); and *S* is the cross-sectional area of the sample in square meters (m^2^).

In addition, the I-V curve was obtained at a scan rate of 0.1 V/s, and the impedance measurements were conducted over a frequency range of 0.1 Hz to 100 kHz.

### 2.6. Contact Impedance of PSPP Hydrogel with Skin

The hydrogel was cut into rectangular samples measuring 10 mm × 10 mm × 2 mm, and two hydrogel samples were placed on the arm with a center-to-center distance of 7 cm, secured using an elastic band. The skin-contact impedance of the PSPP hydrogel was tested using an impedance tester (IM3533-01LCR, HIOKI, Ueda, Japan), with a voltage set at 0.5 V and a scanning frequency range of 0.1 Hz to 1 kHz.

### 2.7. Swelling Ratio and Water Content of PSPP Hydrogel

The hydrogel samples were immersed in deionized water until swelling equilibrium was reached. Afterward, the hydrogels were removed, excess water was blotted off with filter paper, and the mass after swelling equilibrium was recorded. The hydrogels were then placed in a constant temperature drying oven until their mass remained unchanged, and the dry mass of the hydrogel was recorded. The calculations were performed using the following formulas:

$$M_{c}=\frac{M_{w}-M_{d}}{M_{w}}\times 100\%$$

$$E_{SR}=\frac{M_{e}-M_{d}}{M_{d}}\times 100\%$$

where *M_c_* is the water content (%), *E_SR_* is the equilibrium swelling ratio (%), *M_d_* is the mass of the dry hydrogel (g), *M_w_* is the mass of the wet hydrogel (g), and *M_e_* is the mass of the hydrogel at swelling equilibrium (g).

### 2.8. Sensing Properties of PSPP Hydrogel

To develop a strain and motion sensor for human activity monitoring, the prepared PSPP hydrogel was cut into rectangular shapes (10 mm × 2 mm × 2 mm), with each end connected to an electrochemical workstation. The relative change in resistance (Δ*R*/*R*_0_) was calculated using the following formula:

$$\frac{\Delta R}{R_{0}}=\frac{R-R_{0}}{R_{0}}\times 100\%$$

where *R*_0_ represents the initial resistance, and *R* denotes the real-time output resistance.

In strain sensing, the hydrogel was subjected to repeated stretching cycles to measure changes in strain-related electrical signals. For human motion sensing, the hydrogel sheets were secured to various joints of the volunteers using medical tape.

To construct the temperature sensor, the PSPP hydrogel was cut into rectangular samples (10 mm × 2 mm × 2 mm), and the hydrogel sample was connected to an electrochemical workstation to test the voltage response of the PSPP hydrogel at different temperatures.

For physiological signal sensing, the PSPP hydrogel was cut into rectangular samples (10 mm × 10 mm × 2 mm) and fabricated into detachable electrodes. These electrodes were used in conjunction with an elastic bandage and connected to a BIOPAC physiological signal monitoring system to measure ECG, EMG, and EEG signals.

During ECG signal testing, the volunteers were monitored under four different conditions: resting, walking, arm swinging, and squatting. For EMG signal testing, the volunteers performed various gestures, including giving a thumbs up, arm bending, and swinging the arms left and right. Additionally, EMG signals were measured while the volunteers held weights of 1.25 kg, 2.5 kg, and 3.75 kg. During EEG signal testing, volunteers performed a blinking action.

## 3. Results and Discussion

### 3.1. Structural Design of PSPP Hydrogel

**Figure 2:**
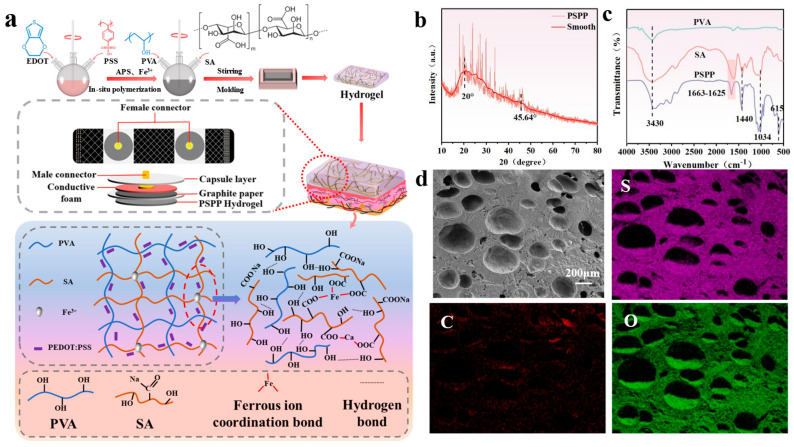
(**a**) The fabrication and structure of multifunctional electrodes; (**b**) the SEM image of the cross-section of the PSPP hydrogel; (**c**) the FT-IR spectra of the PVA, SA, and PSPP hydrogels; (**d**) Fe distribution map in the EDS spectrum of the PSPP hydrogel.

A PVA/SA-based hydrogel, prepared through a cyclic freeze–thaw method, was utilized as a flexible substrate and integrated with PEDOT:PSS as a conductive material to develop a multifunctional flexible sensor capable of detecting temperature, strain, and electrophysiological signals (ECG, EMG, and EEG) (Figure 2a). During the preparation process, cyclic freeze–thawing promoted the crystallization of PVA molecular chains. As the number of freeze–thaw cycles increased, crystalline regions gradually expanded, and crystallinity improved, resulting in a hydrogel with a three-dimensional structure. According to Figure 2b, the peak at 20° may correspond to the crystalline peak of PVA, reflecting the ordered arrangement of PVA molecular chains. Additionally, a small amount of Fe^3+^ ions coordinated with the carboxyl groups (-COOH) in SA, further stabilizing the crosslinked structure. The high electrical conductivity and thermoelectric properties of PEDOT:PSS enabled the sensor to detect temperature variations and mechanical strain [37,38]. The PSPP hydrogel exhibited not only excellent mechanical strength but also multifunctional sensing capabilities, demonstrating high stability and sensitivity in detecting ECG, EMG, and EEG signals. FTIR analysis (Figure 2c) revealed a broad peak at 3430 cm^−1^ in PVA, SA, and PSPP, attributed to the stretching vibrations of hydroxyl groups (-OH). The abundant -OH groups facilitated hydrogen bonding, enhancing the hydrogel’s hydrophilicity. A peak at 1663 cm^−1^ was observed in the PSPP sample, while a peak at 1625 cm^−1^ appeared in the SA sample. These peaks are typically associated with the stretching vibrations of carbonyl groups (-C=O) in carboxylic acids (-COOH). The significant weakening of the 1663 cm^−1^ peak in the PSPP sample might result from the coordination between Fe^3+^ ions and SA [39]. Furthermore, the peak at 1440 cm^−1^ is likely related to the stretching vibrations of C=C bonds in thiophene rings. The peak at 1034 cm^−1^ is attributed to the stretching vibrations of ether bonds (C-O-C), and the peak at 615 cm^−1^ corresponds to the stretching vibrations of C-S bonds, consistent with the structural features of PEDOT:PSS, confirming the presence of thiophene rings in the PEDOT chains. SEM images showed that the PSPP hydrogel possesses a porous structure with relatively large pore sizes (Figure 2d). The porous structure can act as an effective pathway for energy dissipation. When the hydrogel is subjected to external forces, the deformation and collapse of pores absorb partial energy, thereby improving the material’s toughness. The porous structure significantly increases the hydrogel’s specific surface area, providing more active sites for PEDOT:PSS. Additionally, pores serve as channels for ion or electron transport, enhancing the hydrogel’s conductivity. Elemental mapping via EDS confirmed that C, O, and S elements are uniformly distributed throughout the hydrogel, indicating the formation of a homogeneous conductive network by PEDOT:PSS within the hydrogel matrix (Figure 2d). Carbon (C) serves as the primary component of the hydrogel, with its uniform distribution providing a stable matrix for the conductive network. Oxygen (O), derived from hydrophilic groups (e.g., hydroxyl and carboxyl groups) in the hydrogel, contributes to high water content and ionic conductivity. The uniform distribution of O suggests excellent hydrophilicity and ion transport capability. Sulfur (S) originates from PEDOT:PSS, and its homogeneous dispersion indicates well-distributed conductive additives, facilitating the formation of continuous conductive pathways.

### 3.2. Mechanical Properties of PSPP Hydrogel

**Figure 3:**
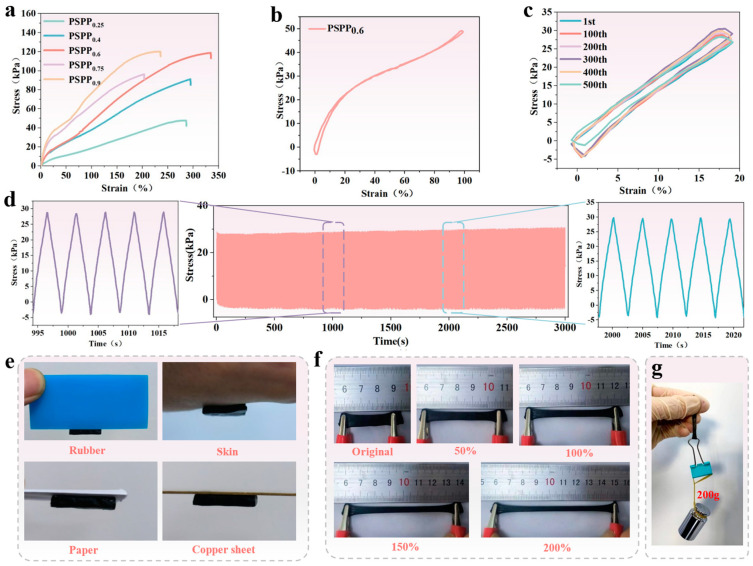
(**a**) Stress–strain curve of PSPP hydrogel. (**b**) Stress–strain curve of PSPP_0.6_ hydrogel after 10 cycles of strain. (**c**) Stress–strain curve of PSPP_0.6_ hydrogel after 500 cycles at 20% strain. (**d**) Stress stability of PSPP_0.6_ hydrogel after 500 cycles at 20% strain. (**e**) Hydrogel can be attached to rubber, skin, paper, and copper sheet. (**f**) Stretching optical images of PSPP hydrogels at different strains; (**g**) demonstrating the ability to lift a 200 g weight.

The effect of PEDOT:PSS content on the mechanical properties of PVA/SA-based hydrogels was investigated. The results showed that a small amount of PEDOT:PSS significantly improved the tensile strength and elongation at break of the hydrogel (Figure 3a). As the PEDOT:PSS content increased, the mechanical properties of the hydrogel continued to improve. This enhancement is likely attributed to hydrogen bonding interactions between PEDOT:PSS molecules and PVA/SA, which strengthened molecular chain entanglement and crosslinking, thereby enhancing the material’s toughness and tensile strength. However, when the PEDOT:PSS content further increased, the elongation at break decreased, primarily due to elevated water content (which hinders the formation of a “viscoelastic structure” by PEDOT:PSS), leading to the dilution of the network structure and reduced crosslinking density between polymer chains. Among the tested samples, the PSPP_0.6_ hydrogel exhibited optimal performance in terms of stress (118.8 kPa) and strain (334%). Further cyclic tests were conducted on the PSPP_0.6_ hydrogel under 100% strain for 10 cycles (Figure 3b) and 20% strain for 500 cycles (Figure 3c) to evaluate its stability and hysteresis. The results demonstrated low strain hysteresis, stable strain response (Figure 3d shows nearly unchanged stress after 500 cycles), and excellent fatigue resistance. The hydrogel could adhere to rubber, skin, paper, and copper plates without detachment (Figure 3e). Additionally, the hydrogel achieved 200% strain and lifted a 200 g weight (Figure 3f,g), fully demonstrating its outstanding mechanical strength and flexibility.

### 3.3. Electrochemical Performance of PSPP Hydrogel

**Figure 4:**
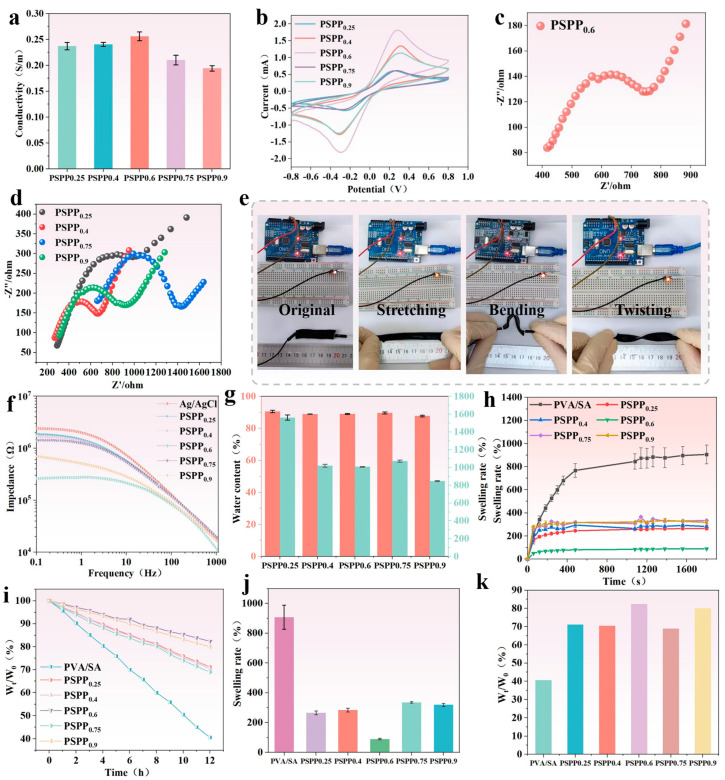
(**a**) I-T curves and corresponding conductivity values; (**b**) I-V curves; (**c**) EIS impedance spectrum of PSPP_0.6_ hydrogel; (**d**) EIS impedance spectrum of other PSPP hydrogels; (**e**) conductivity of the hydrogels under initial, stretched, bent, and twisted states; (**f**) skin-contact impedance of PSPP hydrogels; (**g**) swelling and water content test over 30 h; (**h**) swelling test over 30 h; (**i**) water retention test over 12 h; (**j**) swelling behavior of PSPP hydrogels; (**k**) water retention test over 12 h.

The influence of varying PEDOT:PSS content on the conductivity of PSPP hydrogels was investigated. The conductivity of different hydrogel samples was measured using an electrochemical workstation. The results showed that the PSPP0.6 hydrogel exhibited the highest conductivity of 256 mS/m (Figure 4a). However, as the PEDOT:PSS content further increased, the conductivity of the hydrogels gradually decreased. This suggests that excessive PEDOT:PSS may lead to uneven dispersion within the hydrogel network, preventing the formation of effective and continuous conductive pathways, thereby limiting electrical performance. Cyclic voltammetry (CV) tests (Figure 4b) revealed that PEDOT:PSS participates in energy storage through redox reactions. The area enclosed by the CV curve correlates with the redox capability of the hydrogel—the larger the area, the more favorable the charge–discharge reactions [39]. Among the samples, PSPP_0.6_ demonstrated the largest CV curve area, indicating optimal energy storage characteristics. Electrochemical impedance spectroscopy (EIS) analysis (Figure 4c,d) showed that the PSPP_0.6_ hydrogel exhibited the lowest contact resistance, suggesting higher charge transfer efficiency. The diffusion characteristics observed in the low-frequency region further confirmed the excellent charge transfer capability of this hydrogel in practical applications. Additionally, skin interface impedance tests demonstrated that the PSPP hydrogels consistently exhibited lower impedance values compared to traditional Ag/AgCl gel electrodes, with PSPP_0.6_ showing the lowest contact impedance, highlighting its superior electrical compatibility with human skin (Figure 4f). Notably, the PSPP hydrogels maintained high conductivity under deformations such as stretching, bending, and twisting and were capable of stably powering an LED light (Figure 4e), fully demonstrating their potential for applications in flexible electronic devices.

### 3.4. Swelling Behavior and Water Content of PSPP Hydrogel

The PSPP hydrogel exhibits a highly porous structure capable of storing significant amounts of water, achieving an equilibrium water content of over 85% (Figure 4g). This high water content imparts excellent hydrophilicity and flexibility to the hydrogel, significantly enhancing its skin affinity and ensuring stable contact with the skin. As the PEDOT:PSS content increases, the swelling ratio of the hydrogel decreases notably. This may be attributed to the hydrogen bonding interactions within PEDOT:PSS, which enhance the crosslinking density of the hydrogel, resulting in a tighter molecular network structure that limits water absorption. Further testing reveals that PSPP hydrogels demonstrate low swelling rates over 30 h and minimal water loss over 12 h (Figure 4h,i). Particularly, the PSPP_0.6_ hydrogel exhibits minimal swelling and water loss, indicating superior structural stability (Figure 4j,k).

### 3.5. Strain Sensing of PSPP Hydrogel

**Figure 5:**
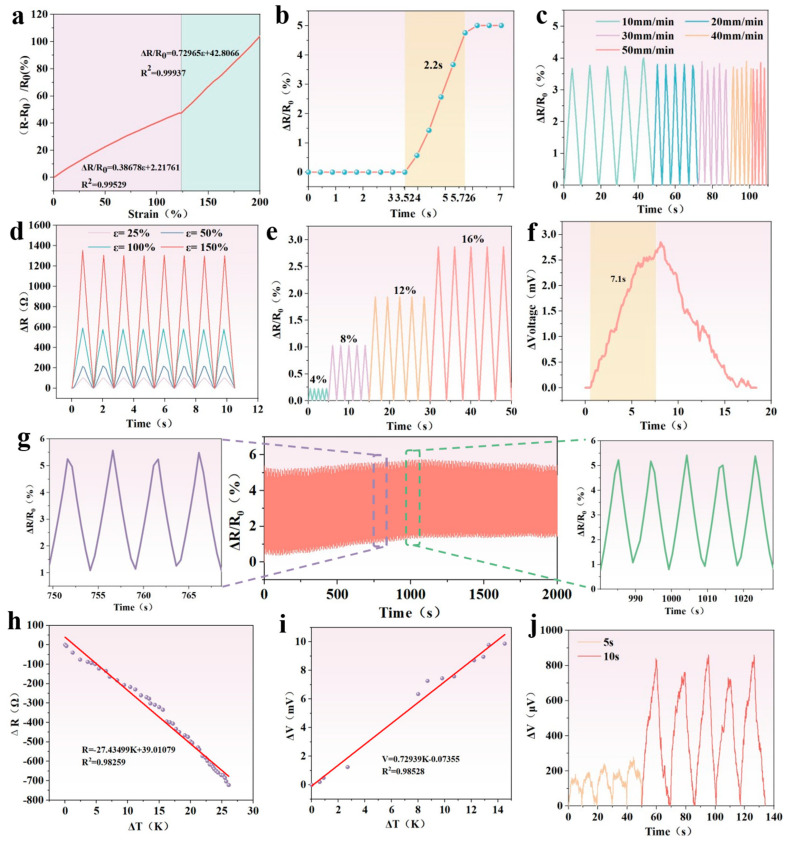
(**a**) Linear fitting of the hydrogel under different strains; (**b**) response time of the hydrogel under 10% strain; (**c**) strain response of the hydrogel under 30% strain at different stretching speeds; (**d**) resistance change in the hydrogel under strains of 25%, 50%, 100%, and 150%; (**e**) strain response of the hydrogel under small strains; (**f**) response time when the irradiation time is 10 s; (**g**) cyclic strain response of the hydrogel under 20% strain for 500 cycles (20 mm/min); (**h**) resistance–temperature fitting curve of the hydrogel with a sensitivity coefficient of −27.43 Ω/K; (**i**) voltage–temperature fitting curve of the hydrogel with a sensitivity coefficient of 0.729 mV/K; (**j**) voltage variation in the hydrogel under different durations of light exposure.

The PSPP hydrogel was fabricated as a strain sensor, and its strain response characteristics were evaluated. As shown in Figure 5a, the resistance change in the sensor exhibits a linear relationship with strain in the ranges of 0–120% and 120–200%, respectively, demonstrating its ability to precisely detect strain through resistance variation. The gauge factors (GFs) for these two ranges are 0.39 and 0.73. To assess the sensor’s response capability, a 10% strain was applied, briefly maintained, and then rapidly released to its initial state (Figure 5b). The results indicated a response time of 2.2 s, showcasing its rapid response performance. Additionally, Figure 5c shows that the strain response signal intensity is insensitive to variations in stretching speed, highlighting the independence of strain response from testing frequency. The sensor’s high sensitivity enables it to accurately capture strain signals and detect lower strain thresholds. Figure 5e demonstrates good periodic response stability under small strains, with the sensor capable of detecting micro-strains as low as 4%. Even under larger strains, the sensor maintains stable response behavior (Figure 5d). The hydrogel material exhibits low strain hysteresis, resulting in minimal resistance hysteresis during loading and unloading processes (Figure 3b). To further evaluate the long-term stability of the sensor’s response signals, 500 loading–unloading cycles were performed on the PSPP hydrogel immersed in glycerol (Figure 5g). The results confirmed that resistance changes remain stable throughout the cyclic testing, indicating excellent cyclic strain robustness. In summary, the PSPP hydrogel strain sensor demonstrates high sensitivity, low hysteresis, and outstanding stability, making it suitable for high-precision strain detection applications.

### 3.6. Temperature Sensing of PSPP Hydrogel

The temperature-sensing properties of PEDOT:PSS originate from enhanced carrier hopping effects and tunneling effects within individual grains and between adjacent nanosheets under thermal stimulation [32]. At room temperature, charge carrier mobility in PEDOT:PSS composites is relatively constrained. However, as the temperature increases, electrons in the valence band are excited and transition to the conduction band, leading to increased conductivity. Additionally, within PEDOT:PSS materials, carrier movement from the hot end to the cold end generates a potential difference, creating a counter flow of charges. When the thermal motion of the charges reaches dynamic equilibrium with the internal electric field, a stable thermoelectric potential is established [35]. Based on these mechanisms, two types of temperature sensors were developed using the PSPP hydrogel: resistance-responsive and voltage-responsive sensors.

As shown in Figure 5h,i, the resistance decreases monotonically with an increasing temperature difference, while the voltage increases monotonically. The sensitivity coefficients for the two sensors are −27.43 Ω/K and 0.729 mV/K, respectively. These sensors exhibit stable cycling performance and output consistent voltage signals under varying temperature gradients, demonstrating excellent thermal response stability. Furthermore, at a power of 1 kW/m^2^, the voltage variation in the sensor was tested under different xenon lamp exposure durations (Figure 5j), with a response time of 7.1 s at an exposure duration of 10 s (Figure 5f). As the surface temperature rises, the sensor-generated voltage increases, indicating its ability to leverage photothermal conversion properties to sense solar radiation while maintaining good cyclic stability [29]. These results highlight the potential of the PSPP hydrogel in photothermal temperature-sensing applications.

### 3.7. Monitoring Human Motion with PSPP Hydrogel

**Figure 6:**
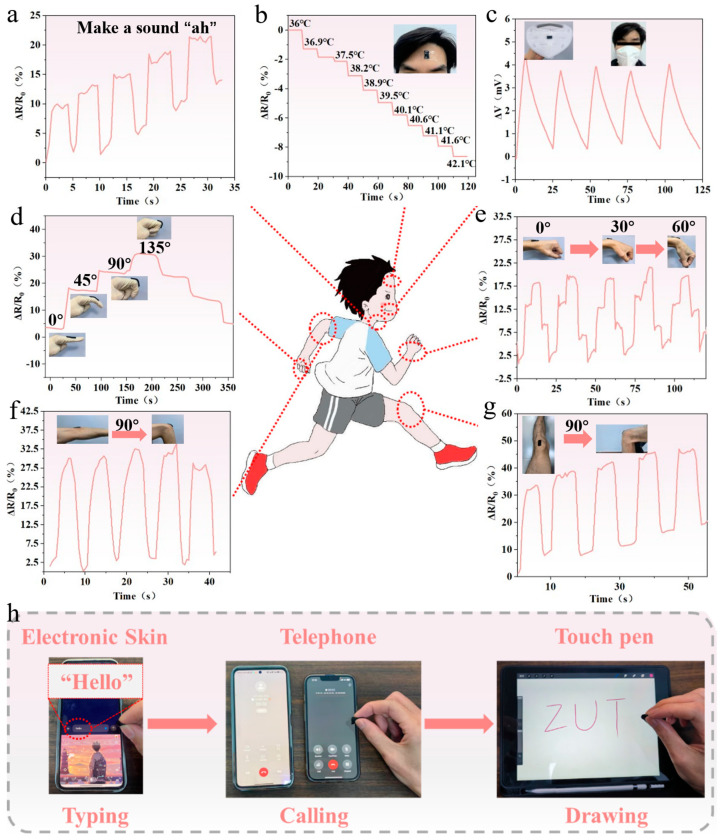
(**a**) Vocalization monitoring. (**b**) Simulated fever scenario. (**c**) Respiration monitoring. (**d**) Finger joint movement. (**e**) Wrist joint movement. (**f**) Elbow joint movement. (**g**) Knee joint movement. (**h**) Typing, dialing, and handwriting detection.

Hydrogel sensors, with their high sensitivity and wide strain-sensing range, exhibit potential for the real-time monitoring of both large deformations (e.g., joint movements) and small deformations (e.g., vocalization) in the human body. When attached to a finger joint, the sensor’s resistance increases monotonically as the finger bending angle grows and decreases as the angle reduces during extension (Figure 6d). This characteristic demonstrates the sensor’s capability to effectively monitor the bending and straightening motions of finger joints and precisely detect bending angles. Additionally, the sensor was mounted on the wrist (Figure 6e), elbow (Figure 6f), and knee (Figure 6g) to measure resistance changes during joint flexion and extension. The experimental results show that the resistance increases monotonically during joint bending and decreases during extension, with the resistance signals exhibiting excellent cyclic stability. This confirms the sensor’s ability to reliably track joint movements. The sensor can also be placed on the throat (Figure 6a) to monitor small strains associated with vocalization. During speaking, the vibrations of the vocal cords induce stretching strain in the sensor, resulting in an increase in resistance. This confirms the sensor’s potential for monitoring vocal cord activity. Given its sensitivity to temperature differences, the sensor was further used for monitoring body temperature. In a simulated fever scenario, the sensor was applied to the forehead (Figure 6b), where resistance decreased as the temperature rose. Furthermore, the sensor can monitor breathing by detecting the temperature difference between the body and the environment (Figure 6c). During exhalation, warm airflow heats the sensor surface, generating stable and repeatable voltage signals. This demonstrates the sensor’s utility as a respiratory monitoring device, capable of reflecting breathing activity through voltage feedback. The PSPP hydrogel sensor demonstrates broad application potential in monitoring human motion and physiological signals, particularly in joint movement, vocalization, temperature monitoring, and respiratory tracking. Moreover, thanks to the hydrogel’s excellent mechanical flexibility, the material shows promise as electronic skin. It could be widely applied to devices such as smartphones to enable tactile interactions like typing, dialing, and handwriting (Figure 6h).

### 3.8. Monitoring Electrophysiological Signals with PSPP Hydrogel

**Figure 7:**
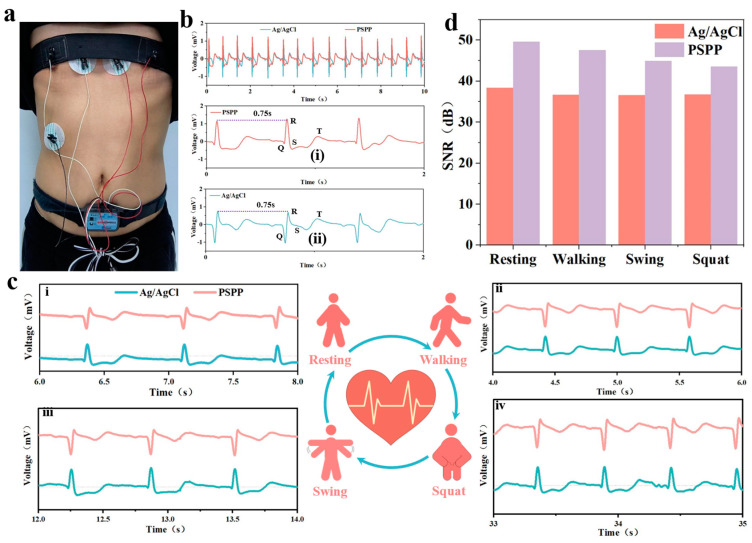
(**a**) Optical image of PSPP hydrogel paired with elastic bandage and disposable Ag/AgCl electrode patch placement. (**b**) Comparison of ECG waveforms between PSPP hydrogel and disposable Ag/AgCl electrodes. (**i**) R-R inter peak of PSPP hydrogel. (**ii**) R-R inter peak of commercial Ag/AgCl gel electrode (**c**) SNR under resting, walking, arm swinging, and squatting states: (**i**) ECG waveform in the resting state, (**ii**) ECG waveform in the walking state, (**iii**) ECG waveform in the arm swinging state, (**iv**) and ECG waveform in the squatting state. (**d**) Signal-to-noise ratio of hydrogel and disposable Ag/AgCl electrodes under four different states.

ECG signals were investigated under different movement states, including static (Figure 7c(i)), walking (Figure 7c(ii)), arm swinging (Figure 7c(iii)), and squatting (Figure 7c(iv)). All ECG curves exhibited high reproducibility and were similar to those obtained with traditional gel electrodes (Figure 7c(ii–iv)). Although some curves showed slight artifacts due to the sliding effect of the electrode on the skin surface, the overall waveforms were smooth and distinguishable. Key ECG features, such as the QRS complex, were clearly visible, and the R-R interval remained stable at 0.75 s, reflecting a normal heart rate of 80 beats per minute, which provides a reliable basis for monitoring heart health [40]. Further analysis revealed that the PSPP hydrogel electrodes provided stable electrical signals across all four movement states. Compared to gel electrodes, the PSPP hydrogel exhibited a higher signal-to-noise ratio (SNR) in all movement states (Figure 7d). In the static state, the SNR of the PSPP hydrogel electrode was 49.68 dB, compared to 38.36 dB for the gel electrode. Under dynamic conditions, the ECG signal quality recorded by the PSPP hydrogel electrode was comparable to that of the gel electrode, clearly capturing multiple ECG features and demonstrating superior signal acquisition capability. Notably, the maximum noise level for the PSPP hydrogel electrode was significantly lower than that of the gel electrode in all tested states. These results further demonstrate the superior performance of the PSPP hydrogel as a bioelectrode and validate its potential for use in wearable devices.

**Figure 8:**
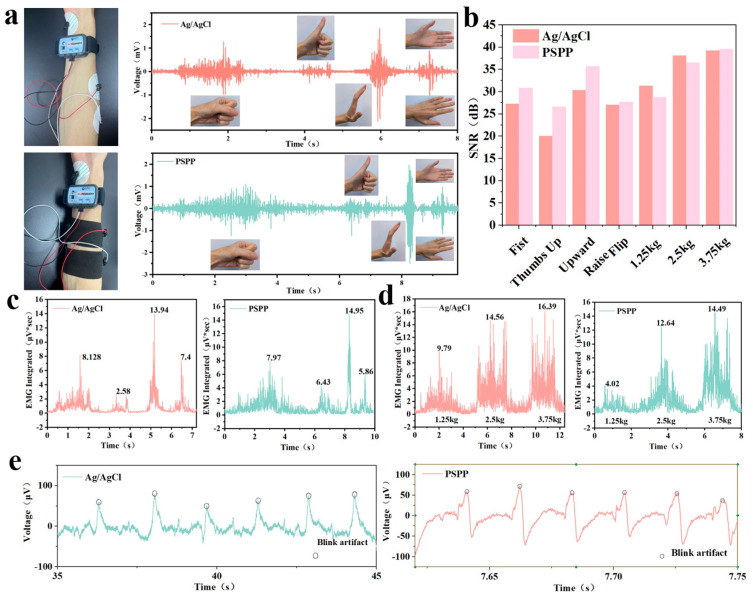
(**a**) Comparison of EMG waveforms between PSPP hydrogel and disposable Ag/AgCl electrodes in different gestures. (**b**) SNR of PSPP hydrogel and disposable Ag/AgCl electrodes during fist clenching, thumbs up, arm raise, hand flipping, and lifting weights of 1.25 kg, 2.5 kg, and 3.75 kg. (**c**) Integrated EMG signals for fist clenching, thumbs up, arm raise, and hand flipping gestures. (**d**) Integrated EMG signals for lifting 1.25 kg, 2.5 kg, and 3.75 kg weights. (**e**) EEG waveform detected by PSPP hydrogel during a blink moment.

Additionally, EMG signals were studied in different postures and while lifting various weights, including fist clenching, thumbs up, arm raises, and hand flipping gestures, as well as lifting weights of 1.25 kg, 2.5 kg, and 3.75 kg [41]. After performing the corresponding actions, all EMG signal curves exhibited prominent peaks and were similar to those obtained with traditional gel electrodes, as shown in Figure 8a. The PSPP hydrogel provided stable electrical signals across seven different motion states. Compared to gel electrodes, PSPP hydrogel showed higher signal-to-noise ratios (SNRs) in different gestures. While it slightly lagged behind disposable Ag/AgCl electrodes when lifting various weights, its overall performance was comparable to that of Ag/AgCl electrodes (Figure 8b). When lifting a 3.75 kg weight, the SNR of the PSPP hydrogel electrode was 39.56 dB, compared to 39.24 dB for the gel electrode. Notably, when integrating the EMG signal analysis, the differences between the PSPP hydrogel and disposable Ag/AgCl electrodes were minimal, with PSPP hydrogel exhibiting comparable EMG signal monitoring ability for various gestures and weight lifting scenarios (Figure 8c,d). Furthermore, PSPP hydrogel also demonstrated potential in EEG monitoring, as it was able to detect EEG waveform changes during blink moments [42]. Upon calculation, the SNR of the PSPP hydrogel for EEG signal monitoring was 10.1123 dB, which was comparable to that of the disposable gel electrodes (Figure 8e).

**Figure 9:**
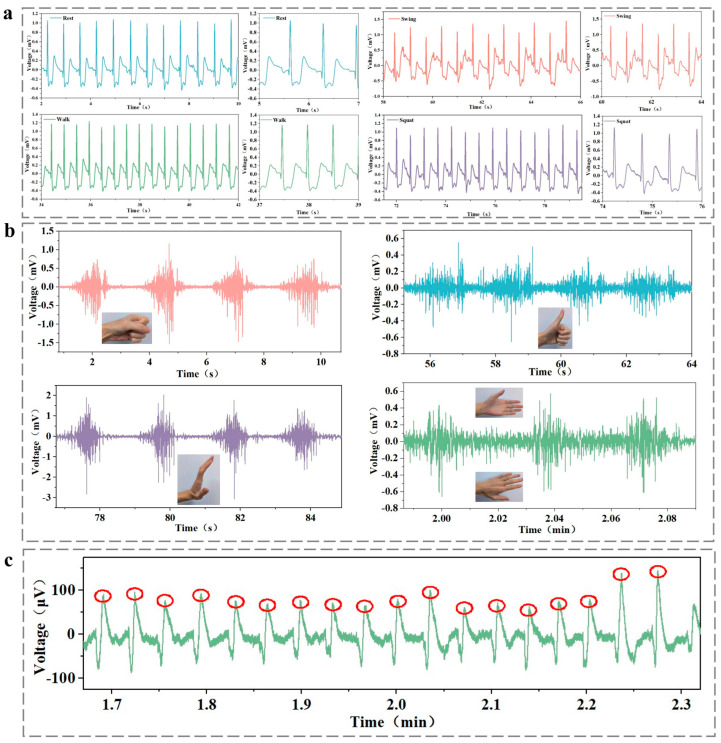
Three months later, PSPP hydrogel (**a**) ECG signals in resting, walking, arm swinging, and squatting states; (**b**) EMG signals in clenching, liking, swinging up, and turning over states; (**c**) EEG signals in blinking states.

Long-term stability is a critical indicator for hydrogel materials in wearable medical devices. To systematically evaluate the durability of the PSPP0.6 hydrogel, samples were stored in a controlled environment (6 °C, 40% relative humidity) simulating real-world usage conditions for three months, followed by multimodal electrophysiological signal monitoring validation (Figure 9). In resting-state ECG monitoring, characteristic QRS complex peaks (R-wave amplitude: 1.02 ± 0.06 mV) were clearly observed, with average heart rate fluctuations controlled within ±3 bpm. During dynamic testing, signal waveforms remained stable during walking and squatting, showing no significant motion artifact interference. Notably, although baseline fluctuations occurred during vigorous arm swinging, QRS complex features were still accurately identified (Figure 9a). In EMG monitoring experiments, the hydrogel demonstrated excellent performance in gesture recognition: a 2.68 mV pulse signal was generated by activation of the abductor pollicis brevis during fist clenching, a 1.15 mV pulse signal was produced by the extensor digitorum muscle group during a “thumbs-up” gesture, and clear pulse signals were also observed during arm raising and twisting motions (Figure 9b). For EEG monitoring, significant alpha wave suppression induced by blinking was observed, and transient potential spikes were reliably captured (Figure 9c). These results confirm the exceptional long-term stability of the PSPP hydrogel in maintaining signal fidelity under diverse physiological conditions.

## 4. Conclusions

The PSPP hydrogel developed in this study demonstrates a wide range of multifunctional sensing applications due to its simple fabrication process, low cost, and low impedance. Experimental results show that the PSPP hydrogel exhibits excellent performance in strain, temperature, and physiological signal detection. As a strain sensor, the PSPP hydrogel is highly sensitive, capable of detecting small strains as low as 4%, and maintains stable performance over a strain range of 0–200%. Its response time is 2.2 s under a 10% strain, making it suitable for monitoring various dynamic activities, from large strains in human joints to small strains in sound vibrations. As a temperature sensor, the PSPP hydrogel demonstrates sensitivities of −27.43 Ω/K and 0.729 mV/K in different modes, showing excellent photothermal conversion properties and meeting the requirements for body temperature and respiration detection. Additionally, the hydrogel supports tactile interaction, making it applicable in activities such as typing, dialing, and writing. In physiological signal monitoring, the PSPP hydrogel performs comparably to disposable Ag/AgCl electrodes in ECG, EMG, and EEG signal detection, with good biocompatibility and stability, making it ideal for continuous health monitoring. Overall, the PSPP hydrogel not only possesses excellent multimodal sensing capabilities but also has a low production cost (less than 0.5 RMB per piece) and a simple fabrication process. It provides a solid material foundation and technical support for the application of flexible sensors in wearable devices, health monitoring, and human–machine interfaces.

Future hydrogels will advance toward enhanced environmental adaptability, functional integration, intelligent sensing, and clinical practicality [43,44,45]. Through material innovation and interdisciplinary technological convergence (e.g., nanotechnology, artificial intelligence), hydrogels are poised to become core components of next-generation intelligent health monitoring systems, driving breakthrough advancements in fields such as medical diagnosis, sports science, and human–machine interaction.

## Data Availability

Data are contained within the article.
